# P02.44. A pilot crossover trial of acupoint injection for treating primary dysmenorrhea

**DOI:** 10.1186/1472-6882-12-S1-P100

**Published:** 2012-06-12

**Authors:** M Chao, C Wade

**Affiliations:** 1 Univ of California, San Francisco, Osher Center for Integrative Med, San Francisco, USA; 2 Institute of East West Medicine, New York City, USA

## Purpose

Primary dysmenorrhea is highly prevalent among adolescent women and a major cause of activity restriction. Standard pharmaceuticals for dysmenorrhea have side effects that may limit their use. Acupoint injection of vitamin K has been used to treat dysmenorrhea at the Obstetrics & Gynecology Hospital in Shanghai, China since at least 1985. The purpose of this study was to examine feasibility and acceptability of acupoint injection of vitamin K1 for primary dysmenorrhea among US women and to assess preliminary data on efficacy.

## Methods

We conducted a crossover trial where study participants were randomized to receive either 1) vitamin K1 injection in the acupoint Spleen-6 at the start of their menstrual cycle followed by a saline injection in a non-acupoint two months later or 2) saline in a non-acupoint followed by vitamin K1 in Spleen-6 two months later. Pain intensity was measured before and after each treatment.

## Results

A total of 18 women with primary dysmenorrhea (mean age = 22 years) enrolled in the study. After participating 94% would still agree to go through with the injection therapy and 77% would come every month if the treatment were available. Based on a 0 to 10 numeric rating scale at baseline and 60 minutes after injection, when receiving vitamin K in Spleen-6, women had an average 2.5 point decrease in pain (4.1 to 1.6). When receiving saline in a non-acupoint, average pain decreased by 1.8 points (4.5 to 2.6).

## Conclusion

Results from this pilot study suggest high acceptability of acupoint injection of vitamin K as a treatment for primary dysmenorrhea among American women. While decreases in pain were observed for both the treatment and control, a trend towards larger pain reduction was seen for vitamin K/Spleen-6 injection. This is consistent with studies conducted in Shanghai, where this treatment protocol was initially developed.

